# Genetics and immunity in the era of single-cell genomics

**DOI:** 10.1093/hmg/ddw192

**Published:** 2016-07-12

**Authors:** Felipe A. Vieira Braga, Sarah A. Teichmann, Xi Chen

**Affiliations:** ^1^Wellcome Trust Sanger Institute; ^2^European Molecular Biology Laboratory, European Bioinformatics Institute (EMBL-EBI); ^3^Cavendish Laboratory, Cambridge University, Cambridge, UK

## Abstract

Recent developments in the field of single-cell genomics (SCG) are changing our understanding of how functional phenotypes of cell populations emerge from the behaviour of individual cells. Some of the applications of SCG include the discovery of new gene networks and novel cell subpopulations, fine mapping of transcription kinetics, and the relationships between cell clonality and their functional phenotypes. Immunology is one of the fields that is benefiting the most from such advancements, providing us with completely new insights into mammalian immunity. In this review, we start by covering new immunological insights originating from the use of single-cell genomic tools, specifically single-cell RNA-sequencing. Furthermore, we discuss how new genetic study designs are starting to explain inter-individual variation in the immune response. We conclude with a perspective on new multi-omics technologies capable of integrating several readouts from the same single cell and how such techniques might push our biological understanding of mammalian immunity to a new level.

## Introduction

Inter-cellular heterogeneity within seemingly homogeneous cell populations has recently emerged as an important source of functional variation within and across samples (1). Over the past few years, technical and methodological developments in the field of single-cell genomics (SCG) have unveiled new biological insights that were previously masked due to measurement approaches that used bulk samples of cells (1,2). By studying gene expression at the single-cell level, one can estimate both the frequency and the strength of transcriptional bursts (3), reflecting the level of noise in gene expression, that is strong but infrequent transcription bursts lead to more noise than small but frequent bursts (4–6). Differential burst behaviour can exist for genes with similar mean expressions in bulk populations, so that biological differences are missed when only bulk samples are analyzed (3,7,8). This detailed information about gene expression can be extracted for each allele (maternal versus paternal) individually, particularly if full-length transcript RNA-seq methods are used (4,6,9).

Another level of information inherent within single-cell RNA-sequencing data are gene regulatory interactions and networks, which can be inferred from correlations and clustering of gene expression variability across large numbers of single cells (10,11). Furthermore, single-cell RNA-seq data from individual T or B cells allow one to fine map their clonality and lineage through the somatically recombined T- or B-cell receptor sequences in addition to maintaining the expression information of all the other expressed genes. This reveals direct correlations between their clonal origin and functional phenotypes (12), information that is impossible to obtain by direct bulk analysis.

**Figure 1.**
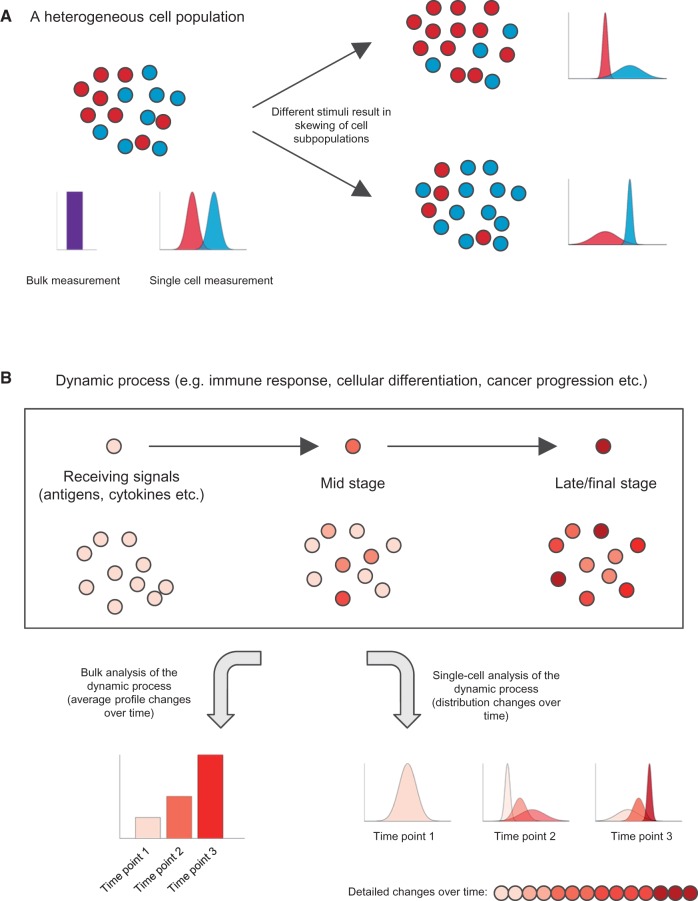
Single-cell measurements retain critical cellular heterogeneity information that is lost by bulk genomics assays. (**A**) Bulk measurements of a cell population cannot distinguish different cellular states. Single-cell analyses can reveal different cell subpopulations and predict/investigate cell skewing upon receiving external stimuli. (**B**) Single-cell measurements provide higher temporal resolution and a more comprehensive overview of a dynamic process.

Beyond the insights mentioned above, a major advantage of SCG methods is that they allow the discovery of new cell states or cell types within a sample (Fig. 1A). SCG methods have frequently led to the discovery of new subtypes of cells without *a priori* knowledge about cell type-specific markers. One of the most commonly investigated systems by SCG technologies has been the mammalian immune system, which consists of a wide variety of cell types responsible to fight infection and cancer. Early single-cell transcriptomic studies showcased the feasibility of identifying distinct cell types from a complex tissue and revealing potential novel markers for specific cell types (13–15). Recent studies further demonstrated that it was possible to uncover new hidden cell subpopulations within very similar cells (16–20). Examples include steroidogenic mouse T helper 2 cells (16), mouse Th2 developmental stages (17), different subpopulations within human ILC3 cells (20), mouse Th17 cells (18), the highly divergent subpopulations of mouse invariant natural killer T (iNKT) cells (21), and most recently, three cellular states during mouse CD4+ T-cell activation (22).

In this review, we will address how new developments in SCG are changing our understanding of biology, with a specific focus on the immune system. The immune system displays tremendous genetic and environmentally determined inter-individual variation and has a central role in determining human health, so is a fertile area for the application of SCG methods.

### Massively parallel single-cell sequencing: what it tells us so far?

In recent years, the field of SCG has advanced rapidly and revolutionized our view of many biological processes. Due to the development of both single-cell capture technologies and whole genome/transcriptome amplification methods, it is now feasible to interrogate the genome and quantify gene expression from single cells by next generation sequencing (NGS). The first step in a typical SCG experiment is to capture individual single cells. This can be done by traditional fluorescence-activated cell sorting of single cells into individual wells of 96- or 384-well plates, or by using microfluidic platforms such as the Fluidigm C1 or microdroplet technologies (23–25). Once individual cells are captured, the cells are lysed, and the genomic DNA or mRNA is amplified by specific protocols to make NGS libraries for sequencing. For single-cell genome sequencing, see two recent reviews by Huang *et al* (26) and by Gawad *et al* (2); for single-cell RNA sequencing (scRNA-seq), see a recent review by Kolodziejczyk *et al* (1).

The genome is usually considered stable during development due to the low error rate of DNA replication (27,28). However, errors do occur, and over sufficient cell divisions, genomic heterogeneity within the same organism due to these somatic mutations—also known as genetic mosaicism—are expected. This genomic heterogeneity can have profound influences on both unicellular and multicellular organisms. Although current genome-wide association study (GWAS) has been very successful in identifying genetic variants that are responsible for many human diseases, most of the discoveries are based on studies conducted at the level of the tissue or individual (29,30). The genetic differences among individual cells are largely neglected and cannot be observed when taking the average signal from a bulk population. However, the genetic heterogeneity of different cells is important in many complex traits, such as cancer (31,32). It is likely that single-cell DNA sequencing, as well as other SCG technologies, will shed light on the role of somatic mutations in the coming years.

Single-RNA sequencing has provided many new insights into immunity in the past few years. For instance, Mahata *et al* investigated the gene expression profiles of many individual mouse T helper 2 (Th2) cells and identified a novel subgroup of Th2 cells that produce the steroid pregnenolone, indicating that this specific Th2 subtype contribute to immune homeostasis via steroidogenesis (16). In a more recent study of the same group, Proserpio *et al* pinpoint the relationship between cellular proliferation and differentiation during *ex vivo* Th2 differentiation and *in vivo* malaria infection, revealing three distinct cell states with different cytokine secretion and proliferation rates. Specifically, the cytokine-secreting T cells proliferate twice as fast as activated cells that do not express cytokines (22).

Gaublomme *et al* studied the heterogeneity of gene expression during the differentiation of Th17 cells in mice. They used both *in vivo* Th17 cells from central nervous system (CNS) and lymph nodes (LN) from an experimental autoimmune encephalomyelitis model of autoimmunity along with *in vitro* differentiated Th17 cells. Data from scRNA-seq indicated that Th17 cells span a spectrum of cellular states with distinct gene expression signatures; furthermore, novel regulatory factors important for Th17 self renewal were identified (18). In a later study, Björklund *et al* investigated the heterogeneity of human innate lymphoid cells (ILCs) by performing scRNA-seq on hundreds of CD127+ ILCs and natural killer (NK) cells from human tonsils (20). Clustering the transcriptomes of 648 cells revealed four distinct populations of cells that correspond to four known ILC populations: ILC1, ILC2, ILC3 and NK cells. More interestingly, the ILC3 group appeared to be very heterogeneous, and further clustering on 1,958 annotated immune genes separated ILC3 cells into three different subpopulations, two out of which have never been described in humans before (20).

Recently, Engel *et al* combined scRNA-seq with bulk H3K27ac ChIP-seq data to investigate the heterogeneity of mouse iNKT subsets (NKT0, NKT1, NKT2 and NKT17). Distinct gene expression programs were identified for different iNKT subsets. More importantly, a substantial difference between cells from within the same subsets was prominent, especially in the NKT2 subset. Interestingly, one particular enhancer, HS V, from within the classical Th2 locus control region (33,34), was identified as a key enhancer for the expression of the cytokine IL-4 in NKT2 cells (21). These studies beautifully illustrate how cells within seemingly homogeneous population can be quite different. Indeed, even among well-defined cell types, new cell subpopulations can often be found.

### Human genetic variation of the healthy immune response

Despite living in an environment, full of life threatening microorganisms, our immune system ensures that humans thrive on our planet. The essential role of the immune system is illustrated by the devastating effects of primary and acquired immunodeficiencies that can lead to persistent infection, cancer susceptibility and even death (35). Uncontrolled immune responses against self (autoimmunity) can lead to extensive tissue destruction and complex clinical manifestations (36). Primary immunodeficiencies are normally associated with one (or a few) well-defined genetic mutations (37), while autoimmune disorders are complex multifactorial diseases where both environment and genetics play a role (36). Despite several single-nucleotide polymorphisms (SNPs) associated with autoimmunity, only a small fraction of carriers develop autoimmunity (38).

The genotypic and phenotypic diversity present in autoimmune disorders is also present in the immune response of ‘healthy’ individuals (39–41). Using a cohort of twin siblings, Brodin *et al* (42) have estimated that the inter-individual variation of CD8 T-cell and B-cell subpopulations is mainly driven by the environment, while CD4 T cells have shown a high degree of heritability (42). Eosinophils, neutrophils and some NK cell features have also shown a high degree of heritability, while monocytes have shown a low heritability. Orru *et al* (43) genotyped a non-twin cohort and analysed more than 200 immune traits, identifying a variable degree of heritability depending on the analysed trait. An especially high degree of heritability was found in CD4 T regulatory cells (Tregs) (43). Tregs are responsible for regulating the extent of the immune response and so play a major role in diseases like cancer and autoimmunity. Therefore, understanding the genetic basis of immune system variation in healthy individuals can improve our understanding of how diseases develop (44).

The studies investigating human immune variation mentioned above have primarily made use of flow cytometry to define populations and analyse specific protein expression in thousands of single cells. Despite their tremendous contribution to our understanding of immune variation, there are limitations to this technology. The number of parameters analysed tends to be small, and they must be selected *a priori*, generating an ascertainment bias. Genomic technologies offer an alternative to overcome such biases. Dimas *et al* (45) analysed primary fibroblasts, Epstein-Barr virus–immortalized B cells (lymphoblastoid cell lines or LCLs), and T cells from the same individual and identified cell-type specific regulatory mechanisms. By combining genotyping with unbiased whole cell transcriptome analysis, they were able to identify expression quantitative trait loci (eQTLs) that controlled gene expression in a cell-type specific way (45), establishing a new paradigm for the study of genetic variation in the immune system.

By analysing peripheral blood total CD4+ T cells, Murphy *et al* (46) identified eQTLs regulating expression of IL23R and IL12R, two cytokine receptors essential for an appropriate immune response, and believed to be involved in autoimmune disorders. To minimize the effects related to the environment and the life history of the cells, Raj *et al (*47*)* analysed naive CD4 T cells, and identified several eQTLs in loci associated with autoimmune diseases, such as rheumatoid arthritis and multiple sclerosis, meaning that the relevant genes are differentially regulated by distinct genetic variants, mostly in non-coding regions (47). While resting naive CD4 T cells display a large number of eQTLs, analysis of effector memory CD4 T cells has identified only a few additional eQTLs (48), including variants in the IL23R locus. Regulatory CD4 T cells also display several eQTLs for genes like ENTPD1, FCRL1, and CD52 (49). Despite our knowledge of the role of many of the genes identified in these studies, such as IL23R (50) and CD52 (51), our overall understanding of the immune cell functions of several of these genes is fairly limited. Further investigations are needed to fully clarify the impact of such genetic variants.

Monocytes play an essential role in orchestrating the immune response: they are capable of presenting antigens and secreting cytokines fundamental for effector function and immune cell regulation (52). Several eQTLs have been identified in monocytes (53,54). The variant rs10784774 at the 12q15 locus was shown to regulate a series of genes, with an especially strong effect detected for *LYZ* and CREB1, genes with important functions for bacterial defense and transcriptional regulation of monocytes (55,56). Neutrophils are another subset of cells essential for the direct bacterial destruction and immune cell regulation via secretion of cytokines (57). Hundreds of eQTLs have been identified to regulate gene expression in human neutrophils (58), including several associated with neutrophil function. One example is rs933222, which was shown to be associated with the expression of RAC2, a gene involved in the NADPH oxidase complex, which is essential for generating the oxidative burst necessary for pathogen killing (58,48).

The studies discussed above suggested the dominance of cell-type specific eQTLs, with very few eQTLs shared across multiple cell types. However, a recent study from Peters *et al* (59) analysed monocytes, CD4 T cells, CD8 T cells, B cells and neutrophils from the same donor. Instead of first computing eQTLs for each cell type and then comparing the lists with each other, they used a Bayesian model (eQTL Bayesian Model Averaging, or ‘eQTLBMA’) to integrate the analysis of eQTLs from the five different cell types within the same model (59). They identified around 45% of the detected eQTLs in all five of the cell types analysed. This result is in marked contrast to previous studies where, for instance, only 21.8% of the detected eQTLs were shared between monocytes and B cells (54). This suggests that appropriate study designs and data analysis approaches are crucial for establishing the real impact of genetic variants in immune cells.

### Four dimensions in genomic data analysis

Most genomic studies analyse cells in their steady state. However, many biological processes such as cancer progression, cellular differentiation and immune response are dynamic. In the context of a dynamic process, different cells may behave differently or respond/progress at different speeds (Fig. 1B). One classic example is the development of the nematode *Caenorhabditis* *elegans*. By exploiting the variation and asynchrony in worm development, Francesconi and Lehner identified thousands of eQTLs during a 12-hour developmental period. This provides a deep characterization of the genetic architecture of the worm’s regulation of development (60).

T-cell activation is another dynamic process in which antigen and inflammatory cytokines mediate extensive transcriptional changes. Ye *et al* (61) analysed gene expression in CD4 T cells during polyclonal T-cell receptor activation under neutral or T helper 17 (Th17) polarising conditions. Despite high inter-individual variability in their cytokine responses, this variability did not have high heritability. Cytokine receptors displayed less inter-individual gene expression variability, yet several eQTLs appear to regulate their expression level (61). Importantly, more than half of the eQTLs identified were not detected when only resting CD4 T cells from the exact same donors were analysed—in other words, they were only revealed upon activation/polarisation (47). Hu *et al* (48) also analysed CD4 T cells after polyclonal activation and in addition to several eQTLs associated with gene expression; they identified rs389862, a variant that directly influences the direct proliferative capacity.

To date, thousands of genetic variants that are associated with complex traits have been identified by GWAS and deep sequencing (62). The majority of the genetic variants are located outside protein-coding regions (63,64), indicating that they are involved in the regulation of the gene expression. Hawkins *et al* (65) performed ChIP-seq to analyse the global chromatin state of Th1 and Th2 CD4 T cells, identifying lineage-specific enhancers. Strikingly, they found several SNPs associated with autoimmune diseases overlapping with these enhancers. For example, they discovered that rs10774213, an SNP associated with type 1 diabetes, resides within an enhancer region of the gene *CCND2*, and has a BACH2 binding motif. BACH2 is a transcription factor that regulates effector T- cell differentiation (66), and the enhancer is one of the most prominent super enhancer regions in mouse T cells (67). In fact, autoimmune disease-associated SNPs tend to be enriched in super enhancer regions (67).

Looking forward to the potential of SCG to make contributions to immune genetics, one could imagine that SNPs located in non-coding regions (such as enhancers and super enhancers) (68) might directly affect transcription kinetics, influencing either burst frequency or intensity. The use of single-cell RNA-seq data will be an invaluable tool to address such questions (3). In a typical SCG experiment, many single cells are captured and analysed so that the temporal information is retained within data from a ‘snap shot’ cell sample (69). By ordering the single cells in ‘pseudotime’ based on their gene expression profiles, one can improve temporal resolution of a dynamic biological process such as differentiation and infection (Fig. 1B), without *a priori* knowledge of marker genes (70).

In an effort to use pseudotemporal ordering of single cells to study myoblast differentiation, Trapnell *et al* developed an unsupervised algorithm called Monocle (70). This was one of the first methods to order single cells in ‘pseudotime’ to quantitatively measure the progress of single cells through a biological process. The algorithm revealed interesting findings relating to skeletal myoblast differentiation, including the switch-like inactivation of the key regulator ID1, a sequential wave of transcriptional reconfiguration and eight novel transcription factors that repress differentiation (70). These examples demonstrate the power of single-cell technologies to integrate the analysis of complex cellular states involved in dynamic temporal processes. We anticipate that these techniques will give us further insights into processes such as cell fate decisions during immune responses.

### Future outlook: multi-omic single-cell methods for genetic and transcriptional analysis

Gene expression heterogeneity is commonly observed in many single-cell transcriptome studies, especially in a dynamic process like the immune response where different cells respond to stimuli in slightly different ways or at different speeds. The extent to which this heterogeneity is due to stochastic differences or genetic differences is not clear at the moment. Therefore, integrating genetic and transcriptomic data at the single-cell level is extremely appealing and will help us to link individual genetic variants to individual cell phenotypes. One early study has revealed scQTLs for seven genes where no eQTLs had been identified by whole-tissue experiments (3). In addition, computational efforts are under way to improve the genetic dissection of certain common traits using data from SCG methods (71).

**Figure 2.**
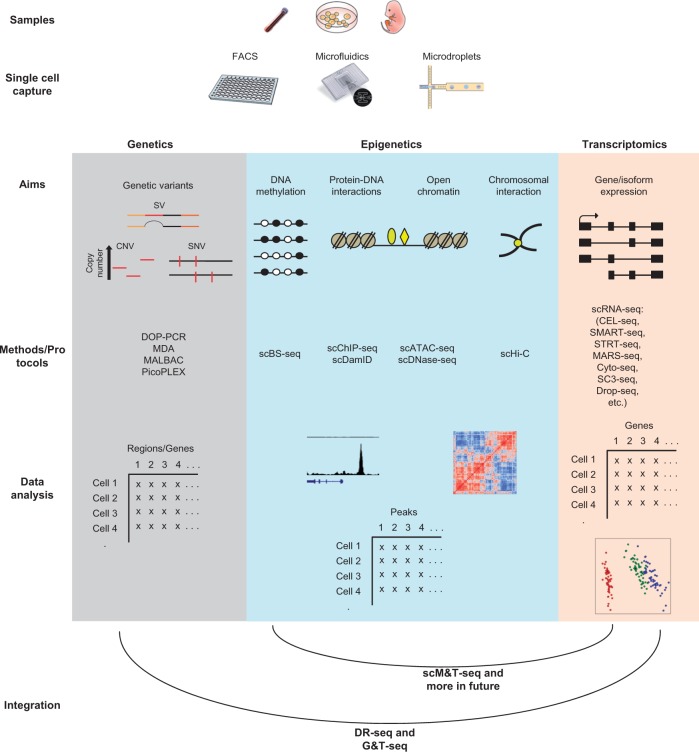
The current workflow of single-cell genomic methods in genetics, epigenetics and transcriptomics, and emerging combined multi-omic technologies from the same single cell. A typical workflow starts with single cell capture through either traditional FACS, microfluidics capture or microdroplets. Then DNA or RNA is prepared for sequencing by different protocols based on the aims of the study and the area of interest (genetics, epigenetics or transcriptomics). Computational analyses are used later on to extract interesting biological information. Recent emerging technologies allow combined genetics-transcriptomics and epigenetics-transcriptomics investigation from the same cell.

At the moment, the development of new methods is a very active area of research in the field of SCG (1,2,72). Many traditional genome-wide assays are now possible at the single-cell level (73–81) (Fig. 2), and combined omic methods from the same cell are now becoming available. Dey *et al* developed a combined genomic DNA and mRNA sequencing (DR-Seq) method (82), and Macaulay *et al* developed a ‘genome and transcriptome’ sequencing (G&T-seq) method (83). Both methods combine whole genome amplification and whole transcriptome amplification to investigate genomic DNA and mRNA from the same cell, which makes the integration of genetic and transcriptional data at the single-cell level feasible. In a more recent study, Angermueller *et al* developed the scM&T-seq method (84), which was based on G&T-seq where a bisulphite convention was performed after the physical separation of genomic DNA from mRNA. This enables the investigation of DNA methylation and gene expression from the same cell.

Multi-omic profiling from the same single cell will usher in an exciting new era of single-cell systems biology, such that hitherto intractable biological questions can be addressed. We predict that more multi-omic methods from the same single cell will be developed in future, providing fundamental insights of a new nature, and revolutionizing the way we investigate basic biological questions.
